# Pre–arrest oral anticoagulants’ impact on cardiac arrest mortality: MIMIC–IV cohort retrospect

**DOI:** 10.3389/fcvm.2025.1599318

**Published:** 2025-11-03

**Authors:** Xianli Nie, Li He, Xiangde Zheng

**Affiliations:** Department of Critical Care Medicine, Dazhou Central Hospital, Dazhou, Sichuan, China

**Keywords:** cardiac arrest, oral anticoagulants, MIMIC database, propensity score matching, mortality

## Abstract

**Background:**

The impact of oral anticoagulant use prior to cardiac arrest (CA) on short-term and long-term all-cause mortality remains largely unknown. This study aimed to explore the association between pre-arrest oral anticoagulant use and both immediate and extended survival outcomes following CA.

**Methods:**

We identified 1,203 adult CA patients from the Medical Information Mart for Intensive Care IV (MIMIC-IV V3.1) database, grouped by prior oral anticoagulant use. Propensity score matching (PSM) was conducted to minimize confounding effects. Adjusted Cox proportional hazards models were applied to account for pre-hospital and hospitalization factors.

**Results:**

Patients in the anticoagulant group demonstrated a significantly higher 28-day survival rate [hazard ratio (HR) 0.28; 95% confidence interval (CI) 0.22–0.37; *P* < 0.001]. After PSM, 120 patients were assigned to the anticoagulant group and 130 to the non-anticoagulant group. In the matched cohort, patients in the anticoagulant group continued to demonstrate improved 28-day survival compared to the non-anticoagulant group (HR 0.40; 95% CI 0.27–0.60; *P* < 0.001). Consistent survival benefits were observed at 90, 180, and 365 days. Subgroup analyses further supported these findings.

**Conclusion:**

Pre-arrest oral anticoagulant use may be associated with improved survival outcomes in CA patients.

## Introduction

Cardiac arrest (CA) is a critical end-stage syndrome characterized by an abrupt cessation of cardiac function, often triggered by specific abnormalities like ventricular arrhythmia, asystole, or pulseless electrical activity, all of which disrupt effective circulation and significantly increase the risk of death (1). Data indicate that approximately 420,000 people experience out-of-hospital cardiac arrest (OHCA) annually in the United States, with a survival-to-discharge rate of less than 10% for OHCA and under 20% for in-hospital cardiac arrest (IHCA) (2, 3). Current clinical guidelines prioritize management strategies focused on controlled ventilation with targeted CO₂ levels and neuroprotective measures to reduce neurological damage from hypoperfusion. Despite these advancements, CA patient survival remains below 40% within 30 days post-hospital discharge, with high long-term mortality rates. This underscores substantial progress in treatment success, yet highlights the need for further improvements, particularly in post-resuscitation care involving blood pressure regulation, fluid balance, and coagulation management (4, 5).

The slowing or cessation of blood flow following CA naturally increases the risk of thrombosis, likely due to underlying pathophysiological characteristics (6). However, during resuscitation, cardiopulmonary resuscitation (CPR) often leads to rib fractures and chest wall trauma, resulting in bleeding or even hemorrhage, complicating the management of coagulation function in these patients (6). For individuals with paroxysmal atrial fibrillation—where flow disturbances are transient but the risk of arterial thrombosis is elevated—anticoagulation therapy has been shown to significantly improve both short- and long-term outcomes (7). Similarly, for successfully resuscitated CA patients with heightened coagulation activity, it remains unclear whether oral anticoagulation can enhance short- and long-term survival rates.

This study aims to investigate the potential impact of oral anticoagulants on short-term and long-term mortality in CA patients following hospital discharge.

## Patients and methods

### Study design

This retrospective study utilizes data from the Medical Information Mart for Intensive Care IV (MIMIC-IV), version 3.1—a comprehensive electronic database containing clinical information on over 190,000 patients and 450,000 hospitalizations at Beth Israel Deaconess Medical Center (BIDMC) in Boston, Massachusetts, USA, from 2008 to 2019. The MIMIC-IV database is organized into four main sections: Emergency Department, Admissions, Intensive Care Unit, and Follow-up (8–10). Access to this database requires prior certification of CITI training or verification of sample-only studies (Xiangde Zheng: 65831141).

### Inclusion and exclusion criteria

The inclusion criteria for this study were: (a) all patients diagnosed with cardiac arrest (ICD-9 code 427.5; ICD-10 codes I469, I468, and I469); and (b) age over 18 years.

**Figure 1:**
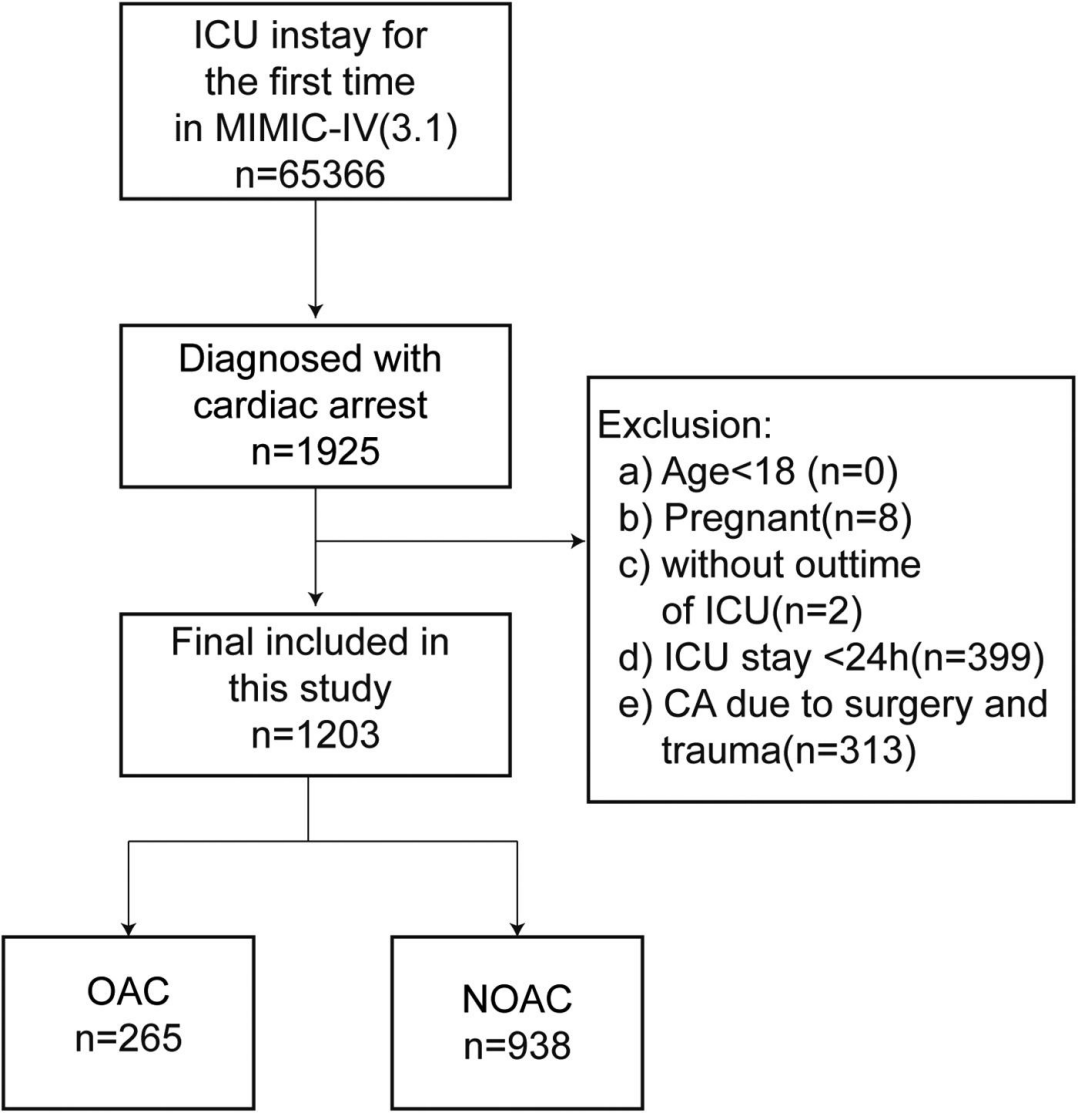
Flow chart of patient selection. OAC, oral anticoagulants; NOAC, no oral anticoagulants.

The exclusion criteria were: (a) pregnancy; and (b) ICU stay of less than 24 h; (c) Trauma or surgery related CA. The inclusion-exclusion process is illustrated in Figure 1.

### Variable extraction

Data extraction was performed using Navicat Premium (version 16.1.15) and SQL. The study examined a range of variables, categorized as follows:

1. Demographic characteristics: Age, sex, body mass index (BMI), and race.
2. Co-morbidities: Previous diagnoses, including myocardial infarction, congestive heart failure, peripheral vascular disease, cerebrovascular disease, chronic pulmonary disease, rheumatic disease, peptic ulcer disease, mild liver disease, severe liver disease, dementia, paraplegia, renal disease, malignant cancer, metastatic solid tumor, atrial fibrillation/flutter, pulmonary embolism, cardiac shock, ventricular arrhythmia and diabetes.
3. Vital Signs: Heart rate (HR), systolic blood pressure (SBP), diastolic blood pressure (DBP), mean blood pressure (MBP), respiratory rate (RR), temperature (T), and pulse oxygen saturation (SpO₂).
4. Laboratory Indicators: Prothrombin time (PT), partial prothrombin time (PTT), International Normalized Ratio (INR).
5. Oral Medications: CCB (Calcium Channel Blockers), Loop Diuretics, PPI (Proton Pump Inhibitors), Spironolactone, *β*-blockers, Anticoagulants, ACEI (Angiotensin-Converting Enzyme Inhibitors), ARB (Angiotensin II Receptor Blockers), and antiplatelet medications.
6. Vasopressors: Dobutamine, dopamine, epinephrine, norepinephrine, phenylephrine, vasopressin.
7. Invasive Therapies: IABP (Intra-Aortic Balloon Pump), Electric Countershock, Invasive Ventilation, CRRT (Continuous Renal Replacement Therapy), PCI/CABG (Percutaneous Coronary Intervention/Coronary Artery Bypass Grafting), ECMO (Extracorporeal Membrane Oxygenation), Mechanical Support.
8. Thrombolytics and Intravenous Anticoagulants: Thrombolytic medications and IV anticoagulants.
9. Length of Stay (LOS) and Outcomes: ICU length of stay, in-hospital death, ICU mortality, 28-day mortality, 90-day mortality, 180-day mortality, and 365-day mortality.
10. Disease Severity Scores: Acute Physiology Score III (APS III), Charlson Comorbidity Index (CCI), and Sequential Organ Failure Assessment (SOFA).

All biochemical variables were measured at admission, before any treatment interventions. Data for the above variables were extracted from the ICD-9 or ICD-10 codes. Variables with missing values exceeding 20% were excluded from the analysis. For variables with missing values under 20%, data imputation was performed using a random forest method.

### Outcomes

The primary outcomes of the present study were 28-day and 365-day all-cause mortality. The secondary outcomes were 90-day and 180-day all-cause mortality.

### Statistical analysis

The normality of continuous variables was first assessed. For non-normally distributed data, the Wilcoxon rank-sum test was used, and results were presented as median with interquartile range (IQR). Categorical variables were compared using the chi-square test or Fisher's exact test, and results were expressed as frequencies and percentages.

Kaplan–Meier (KM) curves and Cox proportional hazards models were employed to calculate hazard ratios (HR) with 95% confidence intervals (CI), considering *P* < 0.05 as statistically significant. KM curves were used to assess the incidence of both primary and secondary outcomes, stratified by anticoagulant use, with the non-anticoagulant group as the reference. Three Cox proportional hazards regression models were established:

1. Model I: Unadjusted model.
2. Model II: Adjusted for demographic variables, comorbidities and vital signs.
3. Model III: Further adjusted for comorbidities, vital signs, laboratory markers, and other potential confounders.

In Model II, the following variables were included: BMI, gender, race, age, myocardial infarction, congestive heart failure, peripheral vascular disease, cerebrovascular disease, dementia, chronic pulmonary disease, rheumatic disease, peptic ulcer disease, mild liver disease, paraplegia, renal disease, malignant cancer, severe liver disease, metastatic solid tumor, diabetes, cardiac shock, ventricular arrhythmia, atrial fibrillation or flutter, and pulmonary embolism, heart rate, systolic blood pressure, diastolic blood pressure, mean blood pressure, respiratory rate, temperature, and oxygen saturation.

Model III built upon Model II, adding ICU stay time, SOFA score, APSIII score, and additional variables such as Charlson comorbidity index, INR, PT, PTT, CRRT, dobutamine, dopamine, epinephrine, norepinephrine, phenylephrine, vasopressin, ventilation, electric countershock, CCB, loop diuretics, PPI, spironolactone, β-blockers, statins, ACEI, ARB, CPR, ECMO, mechanical support, PCI/CABG, anti-platelet drugs, thrombolytic drugs, and intravenous anticoagulants.

Subgroup analysis was performed to explore whether demographic characteristics and comorbidities influenced the association between oral anticoagulant use and mortality.

We also conducted a propensity score matching (PSM) analysis to balance potential confounders between anticoagulant users and non-users. The caliper for the PSM was set at 0.02 of the standard deviation of the logit of the propensity score, with cases having higher propensity scores prioritized for matching. To better control for confounding bias, all variables except for clinical outcomes (30, 90, 180 and 365-day mortality), ICU stay days, hospital stay days, and safety endpoints (intestinal obstruction, aspiration pneumonia, and refeeding syndrome) were included in the PSM model.

All statistical analyses were performed using STATA software (version 18.0, Stata Corp LLC, USA), with a significance level set at *P* < 0.05.

## Results

### Baseline characteristics

As shown in Figure 1, data were extracted from the MIMIC-IV database for 1,925 ICU-admitted patients who experienced CA during their first ICU stay. A total of 1,203 patients were ultimately included in the analysis after excluding individuals younger than 18, pregnant patients, those with ICU stays of less than 24 h, and cases where CA was due to surgical or traumatic causes.

Before PSM, 265 patients treated with oral anticoagulants were classified into the oral anticoagulant group, while 938 patients without anticoagulant treatment were assigned to the non-oral anticoagulant group. The original cohort displayed a higher mean age, elevated mean BMI, and a higher prevalence of comorbidities, including heart failure, peripheral vascular disease, and chronic kidney disease. Additionally, the cohort had higher mean values for INR, PT, and PTT. Table 1 provides an overview of baseline characteristic differences between the oral anticoagulant group and the non-oral anticoagulant group.

**Table 1:** Baseline characteristics of patients.

Characteristic	Overall		Anticoagulants	Not–anticoagulants	*P* value	SMD	Overall	Anticoagulants	Not–anticoagulants	*P* value	SMD
	*N* = 1,203		*N* = 265	*N* = 938			*N* = 250	*N* = 120	*N* = 130		
Demographic											
Female, *n* (%)	451 (37.5)		103 (38.9)	348 (37.1)	0.600	−0.042	99 (39.6)	46 (38.3)	53 (40.8)	0.700	−0.083
Age	67.2 (56.2–78.8)		71.7 (61.3–81.3)	66.2 (54.8–77.6)	<0.001	0.370	71.2 (61.3˗–81.5)	71.7 (61.3–81.9)	71.0 (61.5–80.2)	0.750	−0.080
BMI	26.7 (21.1–31.9)		28.3 (22.6–32.0)	26.4 (20.6–31.7)	0.016	−0.026	28.3 (23.4–33.0)	28.7 (23.3–32.8)	27.6 (22.7–33.3)	0.413	−0.087
Race, *n* (%)					<0.001					0.756	
Black	119 (9.89)		35.0 (13.2)	84.0 (8.96)		0.112	25.0 (10.0)	12.0 (10.0)	13.0 (10.0)		−0.079
White	653 (54.3)		162 (61.1)	491 (52.3)			164 (65.6)	76.0 (63.3)	82.0 (67.7)		
Other	431 (35.8)		68.0 (25.7)	363 (38.7)		0.180	61.0 (24.4)	32.0 (26.7)	29.0 (22.3)		−0.082
Vital signs											
Heart rate (beats/min, IQR)	81.5 (70.4–95.6)		80.8 (70.8–92.9)	81.9 (70.2–96.1)	0.568	−0.086	82.0 (71.6–95.7)	78.7 (70.6–90.9)	86.7 (72.2–98.5)	0.034	0.072
SBP (mmHg, IQR)	112 (105–124)		111 (105–122)	112 (105–124)	0.440	0.008	113 (105 –23.9)	113 (105–124)	113 (105–125)	0.778	−0.037
DBP (mmHg, IQR)	63.2 (56.1–70.5)		63.1 (56.2–70.5)	63.2 (56.0–70.4)	0.707	0.107	62.9 (9.64)	62.9 (9.91)	63.0 (9.41)	0.940	0.085
MBP (mmHg, IQR)	78.1 (71.9–85.1)		77.9 (71.8–85.1)	78.1 (72.0–85.1)	0.952	0.099	77.5 (71.3–84.3)	77.4 (71.7–85.2)	77.6 (71.2–83.7)	0.753	−0.002
resp rate (breath/min, IQR)	20.3 (17.7–23.7)		19.5 (17.2–22.7)	20.5 (17.8–24.1)	0.001	−0.214	19.7 (17.2–23.2)	19.8 (17.2–22.9)	19.7 (17.2–23.6)	0.498	−0.077
Temperature (°C, IQR)	36.7 (36.3–37.1)		36.8 (36.5–37.0)	36.7 (36.2–37.1)	0.062	0.193	36.8 (36.5–37.1)	36.8 (36.5–37.0)	36.8 (36.6–37.1)	0.454	−0.070
spo2 (%, IQR)	97.7 (96.1–99.0)		97.3 (97.3–98.6)	97.8 (96.1–99.1)	0.059	0.002	97.2 (95.6–98.5)	97.1 (95.9–98.4)	97.2 (95.4–98.6)	0.900	−0.036
Co–morbidities											
Diabetes, *n* (%)		406 (33.8)	98.0 (37.0)	308 (32.8)	0.208	0.076	97 (38.8)	46 (38.3)	51 (39.2)	0.897	−0.045
Myocardial infarct, *n* (%)		393 (32.7)	94 (35.5)	299 (31.9)	0.270	0.095	93.0 (37.2）	46.0 (38.3)	47.0 (36.2)	0.794	0.012
Congestive heart failure, *n* (%)		502 (41.7)	176 (66.4)	326 (34.8)	<0.001	0.069	140 (56.0)	72 (60.0)	68 (52.3)	0.252	0.047
Peripheral vascular disease, *n* (%)		175 (14.6)	50.0 (20.0)	122 (13.0)	0.004	0.203	52.0 (20.8)	26.0 (21.7)	26.0 (20.0)	0.758	0.043
Cerebrovascular disease, *n* (%)		208 (17.3)	51.0 (19.3)	157 (16.7)	0.341	0.031	49.0 (19.6)	23.0 (19.2)	26.0 (20.0)	0.498	−0.013
Dementia, *n* (%)		37.0 (3.08)	9.00 (3.40)	28.0 (2.99)	0.690	0.055	11.0 (4.40)	5.00 (4.17)	6.00 (4.61)	1.000	−0.076
Chronic pulmonary disease, *n* (%)		306 (25.4)	71.0 (26.8)	235 (25.1)	0.566	0.007	73.0 (29.2)	35.0 (29.2)	38.0 (29.2)	1.000	0.001
Rheumatic disease, *n* (%)		41.0 (3.41)	11.0 (4.15)	30.0 (3.20)	0.445	−0.004	8.00 (3.20)	3.00 (2.50)	5.00 (3.85)	0.724	0.001
Peptic ulcer disease, *n* (%)		35.0 (2.91)	8.00 (3.02)	27.0 (2.88)	0.838	0.003	5.00 (2.00)	3.00 (2.50)	2.00 (1.54)	0.673	0.030
Mild liver disease, *n* (%)		180 (15.0)	30.0 (11.3)	150 (15.6)	0.064	−0.133	36.0 (14.4)	16.00 (13.3)	20.0 (15.4)	0.720	−0.065
Paraplegia, *n* (%)		55.0 (4.57)	8.00 (3.02)	47.0 (5.01)	0.187	−0.132	9.00 (3.60)	4.00 (3.33)	5.00 (3.84)	1.000	−0.089
Renal disease, *n* (%)		333 (27.7)	101 (38.1)	232 (24.7)	<0.001	0.244	83.0 (33.2)	42.0 (35.0)	41.0 (31.5)	0.592	−0.045
Malignant cancer, *n* (%)		141 (11.7)	24.0 (9.06)	117 (12.5)	0.132	−0.206	32.0 (12.8)	11.0 (9.17)	21.0 (16.2)	0.129	−0.045
Severe liver disease, *n* (%)		60.0 (4.99)	7.00 (2.64)	53.0 (5.65)	0.054	−0.168	11.0 (4.40)	4.00 (3.33)	7.00 (5.38)	0.543	−0.062
Metastatic solid tumor, *n* (%)		58.0 (4.82)	10.0 (3.77)	48.0 (5.12)	0.420	−1.074	11.0 (4.40)	5.00 (4.17)	6.00 (4.61)	1.000	−0.062
AIDS, *n* (%)		3.00 (0.250)	1.00 (0.380)	2.00 (0.210)	0.526						
Cardiac shock, *n* (%)		254 (16.7)	182 (21.4)	72.0 (10.7)	0.102	0.105	52.0 (20.8)	26.0 (21.7)	26 (20.0)	0.758	0.014
Ventricular arrhythmia, *n* (%)		433 (36.0)	129 (48.7)	304 (32.4)	<0.001	0.427	106 (42.4)	52.0 (43.3)	54.0 (41.5)	0.799	0.033
Atrial fibrillation, *n* (%)		447 (37.2)	186 (70.2)	261 (27.8)	<0.001	0.386	162 (64.8)	75.0 (62.5)	87.0 (66.9)	0.508	−0.041
Acute pulmonary embolism, *n* (%)		51.0 (4.24)	19.0 (7.17)	32.0 (3.41)	0.007	0.156	18.0 (7.20)	9.00 (7.50)	9.00 (6.92)	1.000	0.016
Scores											
Sofa, (IQR)	7.00 (4.00–10.0)		6.00 (4.00–9.00)	8.00 (4.00–11.00)	<0.001	-.0296	7.00 (4.00–10.0)	6.50 (4.00–10.00)	7.00 (3.00–10.0)	0.733	−0.061
Aps III, (IQR)	56.0 (41.0–78.0)		49.0 (41.0–66.0)	58.0 (40.0–82.0)	<0.001	−0.336	52.0 (39.0–71.0)	49.5 (41.5–67.0)	53.0 (38.0–77.0)	0.517	−0.077
Charlson comorbidity index, (IQR)	5.00 (3.00–8.00)		6.00 (4.00–8.00)	5.00 (3.00–7.00)	<0.001	0.336	6.34 (3.94)	6.28 (3.07)	6.39 (2.83)	0.770	−0.058
Laboratory tests											
INR, (IQR)	1.30 (1.10–1.60)		1.50 (1.20–2.00)	1.30 (1.10–1.50)	<0.001	0.296	1.30 (1.20–1.80)	1.40 (1.20–1.85)	1.30 (1.20–1.70)	0.350	−0.092
PT (sec, IQR)	14.2 (12.6–17.7)		16.4 (13.4–21.9)	13.9 (12.4–16.8)	<0.001	0.293	14.6 (12.7–19.4)	15.0 (12.9–20.0)	14.4 (12.6–18.8)	0.356	−0.091
PTT (sec, IQR)	33.9 (28.5–53.2)		41.8 (30.9–68.7)	32.6 (28.0–47.0)	<0.001	0.309	34.2 (28.8–58.8)	35.5 (28.8–59.6)	33.5 (28.8–55.3)	0.783	−0.086
Medications											
CCB, *n* (%)	171 (14.2)		55 (20.8)	116 (12.4)	0.001	0.231	54.0 (21.6)	28.0 (23.3)	26.0 (20.0)	0.542	−0.049
Loop diuretics, *n* (%)	734 (61.0)		212 (80.0)	522 (55.7)	<0.001	0.504	188 (75.2)	89.0 (74.2)	99.0 (76.2)	0.770	−0.085
PPI, *n* (%)	692 (57.5)		174 (65.7)	518 (55.2)	0.002	0.222	166 (66.4)	79.0 (65.8)	87.0 (66.9)	0.894	−0.012
Spironolactone, *n* (%)	81.0 (6.73)		37.0 (14.0)	44.0 (4.69)	<0.001	0.317	26.0 (10.4)	13.0 (10.8)	13.0 (10.0)	0.839	0.035
β blocker, *n* (%)	755 (62.7)		229 (86.4)	526 (56.1)	<0.001	0.694	199 (79.6)	79.0 (65.8)	85.0 (65.4)	0.877	0.001
Antiplatelet, *n* (%)	674 (56.0)		194 (73.2)	480 (51.2)	<0.001	0.473	170 (68.0)	83.0 (69.2)	87.0 (66.8)	0.786	<0.001
ACEI, *n* (%)	289 (24.0)		95.0 (35.9)	194 (20.7)	<0.001	0.365	77.0 (30.8)	41.0 (34.2)	36.0 (27.7)	0.276	0.016
ARB, *n* (%)	86.0 (7.15)		37.0 (14.0)	49.0 (5.22)	<0.001	0.261	27.0 (10.8)	11.0 (9.17)	16.0 (12.3)	0.541	−0.062
Tatins, *n* (%)	593 (49.3)		194 (73.2)	399 (42.5)	<0.001	0.678	164 (65.6)	79.0 (65.8)	85.0 (65.4)	1.000	−0.013
Intravenous Anticoagults, *n* (%)	1,093 (90.9)		254 (98.9)	839 (89.4)	0.001	0.325	236 (94.4)	115 (95.8)	121 (93.1)	0.786	−0.045
Thrombolytic, *n* (%)	152 (12.6)		47.0 (17.7)	105 (11.2)	0.006	−0.234	47.0 (18.8)	23.0 (19.2)	24.0 (18.5)	1.000	0.003
Vasoactive drug											
Dobutamine, *n* (%)	48.0 (3.40)		14.0 (5.28)	34.0 (3.62)	0.217	0.078	12.0 (4.80)	7.00 (5.83)	5.00 (3.84)	0.560	0.083
Dopamine, *n* (%)	150 (12.5)		27.0 (10.2)	123 (13.1)	0.246	−0.062	22.0 (8.80)	13.0 (10.8)	9.00 (6.92)	0.372	−0.053
Epinephrine, *n* (%)	184 (15.3)		35.0 (13.2)	149 (15.9)	0.334	−0.123	32.0 (12.8)	14.0 (11.7)	18.0 (13.9)	0.706	−0.078
Norepinephrine, *n* (%)	620 (51.5)		121 (45.7)	499 (53.2)	0.030	−0.183	116 (46.4)	53.0 (44.2)	63.0 (48.5)	0.527	−0.089
Phenylephrine, *n* (%)	295 (24.5)		59.0 (22.3)	236 (25.2)	0.333	−0.059	55.0 (22.0)	23.0 (19.2)	32.0 (24.6)	0.360	0.039
Vasopressin, *n* (%)	269 (22.4)		42.0 (15.9)	227 (24.2)	0.003	−0.219	45.0 (18.0)	20.0 (16.7)	25.0 (19.2)	0.625	−0.017
Procedure											
IABP, *n* (%)	30.0 (2.49)		10.0 (3.77)	20.0 (2.13)	0.177	0.019	9.00 (3.60)	6.00 (5.00)	3.00 (2.31)	0.319	−0.024
Electric countershock, *n* (%)	133 (11.1)		56.0 (21.1)	77.0 (8.21)	<0.001	0.352	41.0 (16.4)	20.0 (16.7)	21.0 (16.2)	1.000	0.046
Invasive ventilation, *n* (%)	719 (59.8)		118 (44.5)	601 (64.1)	<0.001	−0.400	119 (47.6)	58.0 (47.5)	62.0 (47.7)	1.000	0.033
CRRT, *n* (%)	152 (12.6)		33 (12.5)	119 (12.7)	1.00	−0.072	34.0 (13.6)	14.0 (11.7)	20.0 (15.4)	0.462	−0.826
CPR, *n* (%)	440 (36.6)		121 (45.7)	319 (34.0)	0.001	0.240	126 (50.4)	57 (47.5)	69 (53.1)	0.448	−0.058
ECMO, *n* (%)	24.0 (2.00)		5.00 (1.89)	19.0 (2.03)	1.00	−0.013	5.00 (2.00)	3.00 (2.50)	2.00 (1.54)	0.673	0.040
Mechanical support, *n* (%)	29.0(2.41)		7.00 (2.64)	22.0 (2.35)	0.821	0.028	8.00 (3.20)	5.00 (4.17)	3.00 (2.31)	0.486	−0.093
PCI/CABG, *n* (%)	130 (10.8)		36.0 (13.6)	94.0 (10.0)	0.116	0.047	38.0 (15.2)	16.0 (13.3)	22.0 (16.9)	0.483	0.038
ICU stay time (hours, IQR)	93.6 (50.7–192)		116 (56.4–223)	89.0 (49.9–180)	0.001	0.169	103.9 (56.0–223)	119 (64.4–222)	95.7 (53.7–238	0.355	−0.075

PCI, percutaneous coronary intervention; CABG, coronary artery bypass grafting; CPR, cardiopulmonary resuscitation; CRRT, continuous renal replacement therapy; ECMO, extracorporeal membrane oxygenation; IABP, intra-aortic balloon pump; CCB, calcium channel blocker; PPI, proton pump inhibitor, β blocker, beta-blocker; ACEI, angiotensin-converting enzyme inhibitor; ARB, angiotensin II receptor blocker; Statins, HMG-CoA reductase inhibitors; INR, international normalized ratio; PT, prothrombin time; PTT, partial thromboplastin time; SOFA, sequential organ failure assessment; APS III, acute physiology score III; AIDS, acquired immunodeficiency syndrome; MBP, mean blood pressure; SBP, systolic blood pressure; DBP, diastolic blood pressure; SpO2, peripheral capillary oxygen saturation; SMD, standardized mean difference.

Overall, the majority of baseline characteristics were unevenly distributed between the two groups. After PSM, a final cohort of 250 patients was analyzed, including 120 patients on oral anticoagulants and 130 patients not receiving anticoagulant therapy. The baseline characteristics were well balanced between these two groups, with all variables showing *P*-values above 0.05 and SMD absolute values less than 0.1, as illustrated in Table 1.

### Survival analysis

**Figure 2:**
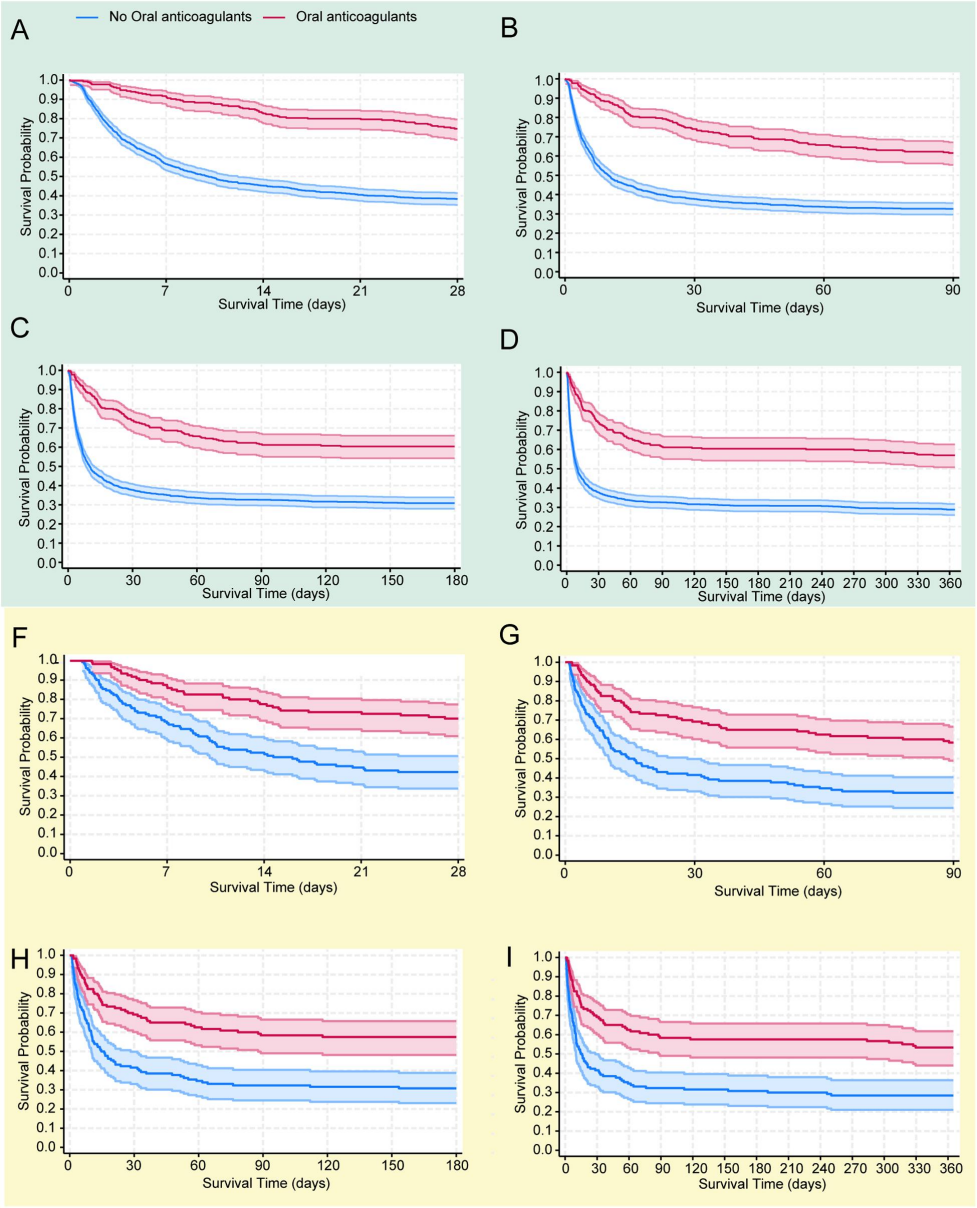
The Kaplan–Meier survival curves show all-cause mortality probabilities for each group at 28 days **(A)**, 90 days **(B)**, 180 days **(C)**, and 365 days **(D)** in the original cohort, and similarly for the propensity score-matched (PSM) cohort at 28 days **(E)**, 90 days **(F)**, 180 days **(G)**, and 365 days **(H)**.

The objective was to examine differences in all-cause mortality between the two groups at various follow-up intervals: 28 days, 90 days, 180 days, and 365 days. In the original cohort, the 28-day, 90-day, 180-day and 365 day mortality rates were significantly lower in the oral anticoagulant group compared to the non-oral anticoagulant group (*P* < 0.05), as shown in Figure 2. Following PSM, KM survival curves confirmed the findings from the original cohort (Figure 2).

**Figure 3:**
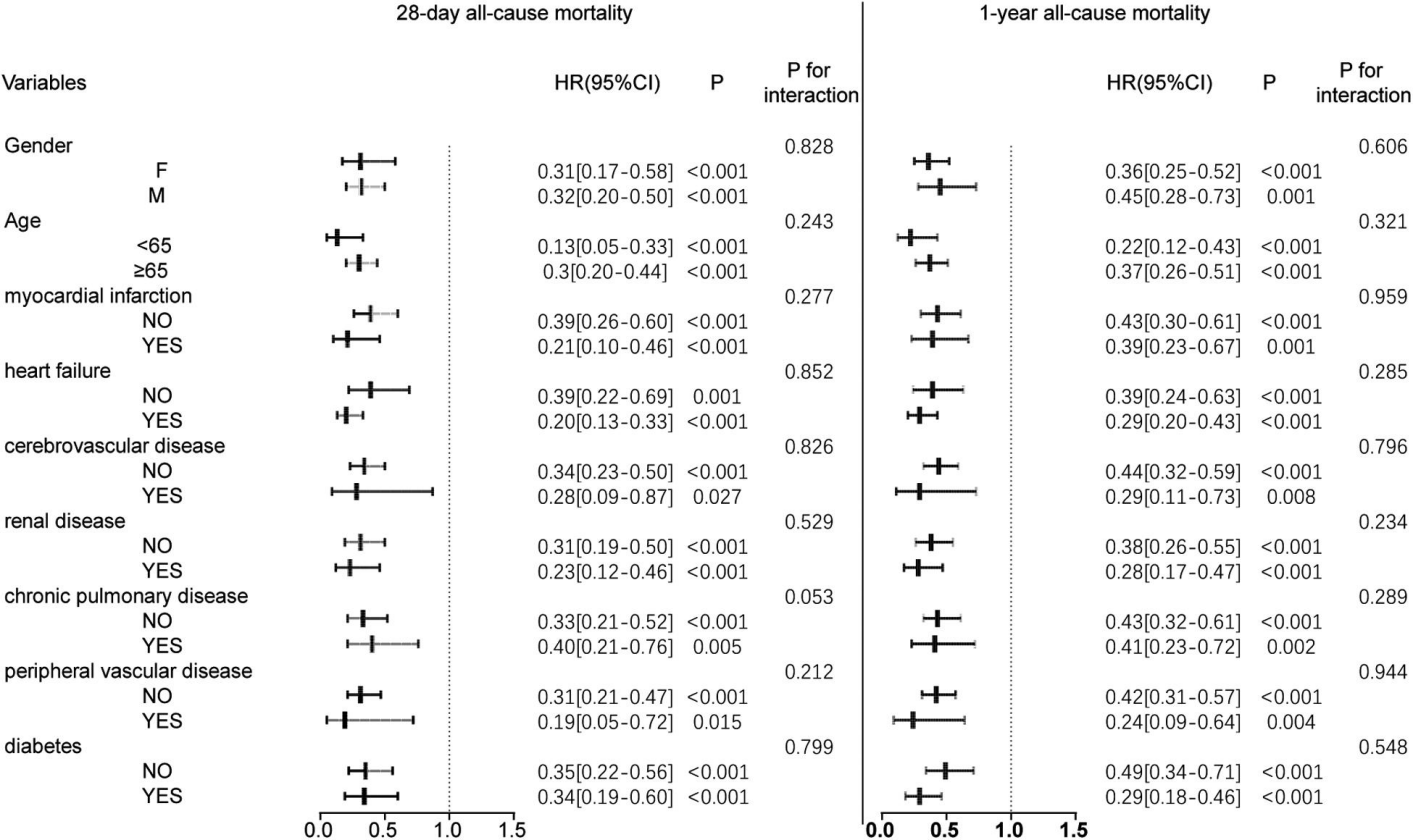
Forest plot of hazard ratios for the primary endpoint across different subgroups. HR, hazard ratio; CI, confidence interval.

### Relationship between oral anticoagulant use and all-cause mortality

To further analyze the association between oral anticoagulant therapy and mortality outcomes, Cox proportional hazards models were applied, with results displayed in Table 2. Univariate Cox regression analysis indicated that oral anticoagulant use was associated with a 72%, 62%, 62%, and 60% reduction in the risk of mortality at 28 days, 90 days, 180 days, and 365 days, respectively, in the original cohort. After adjusting for demographic characteristics, medical ahistory, and baseline vital signs (Table 2), oral anticoagulant use was associated with a 73%, 65%, 66%, and 65% reduction in 28-day, 90-day, 180-day, and 365 day mortality, respectively, compared to the non-oral anticoagulant group.

**Table 2:** Univariate Cox regression analysis.

Events	OR [95%CI]	*P* (Log-rank)	OR [95%CI]	*P* (Log-rank)
28 all-cause mortality	0.28 [0.22–0.37]	<0.001	0.40 [0.27–0.60]	<0.001
90 all-cause mortality	0.38 [0.31–0.47]	<0.001	0.45 [0.32–0.63]	<0.001
180 all-cause mortality	0.38 [0.31–0.47]	<0.001	0.44 [0.31–0.63]	0.008
365 all-cause mortality	0.40 [0.32–0.48]	<0.001	0.46 [0.33–0.64]	<0.001

OR, odds ratio; 95%CI, 95% confidence interval.

In Model III, after further adjustment for laboratory findings and treatment variables, the oral anticoagulant group showed a reduced mortality risk of 64%, 61%, 62%, and 60% at 28 days, 90 days, 180 days, and 365 days, respectively, relative to the non-oral anticoagulant group (Table 3).

**Table 3:** Cox regression analysis of the three models.

Events	Model I		Model II		Model III	
	HR	*P*	HR	*P*	HR	*P*
28 all-cause mortality	0.28 [0.22–0.37]	<0.001	0.27 [0.20–0.35]	<0.001	0.36 [0.24–0.47]	<0.001
90 all-cause mortality	0.38 [0.31–0.47]	<0.001	0.35 [0.27–0.44]	<0.001	0.39 [0.29–0.53]	<0.001
180 all-cause mortality	0.38 [0.31–0.47]	<0.001	0.34 [0.27–0.43]	<0.001	0.38 [0.38–0.51]	<0.001
365 all-cause mortality	0.40 [0.32–0.48]	<0.001	0.35 [0.28–0.44]	<0.001	0.40 [0.33–0.53]	<0.001

HR, hazard ratio; 95%CI, 95% confidence interval.

As shown in Supplementary Table S1, Model III demonstrated the best fit for the 28-day, 90-day, 180-day, and 365 day mortality analyses, with the lowest AIC (Akaike Information Criterion) and BIC (Bayesian Information Criterion) values across these time points (Supplementary Table S1). Additionally, following PSM, the oral anticoagulant group exhibited a significant reduction in all-cause mortality. Specifically, there was a 60% reduction in the risk of death at 28 days, 55% at 90 days, 56% at 180 days, and 54% at 365 days.

### Subgroup analysis

Subgroup analyses were performed to further examine the relationship between oral anticoagulant use and mortality at 28 days and 365 days. The results, presented in Figure 3, show no interaction between the stratification variables and oral anticoagulant exposure (*P* > 0.05 for interaction), indicating that the association between anticoagulant use and mortality was consistent across subgroups.

## Discussion

According to data from this retrospective cohort study using MIMIC-IV database, oral anticoagulant use may be significantly associated with a reduction in all-cause mortality from 28 days to one year following CA.

CA represents the most severe form of this condition, whereby the heart exhibits complete inactivity or markedly inefficient fibrillation, resulting in a slowed or even complete cessation of circulating blood flow. It is one of three principal factors in thrombosis, the others being endothelial damage, slowed or disturbed blood flow, and hypercoagulability. Venous/arterial thrombotic events occurred in 23.5% of consecutive patients with refractory ventricular tachycardia/ventricular fibrillation out-of-hospital cardiac arrest who met the criteria for initiation of extracorporeal cardiopulmonary resuscitation (6). Furthermore, it has been demonstrated that transthoracic cardiopulmonary resuscitation increases the likelihood of traumatic hemorrhage during the resuscitation process after CA. A significant proportion of patients (67.5%) experienced major hemorrhage, while 36.5% had traumatic hemorrhage due to cardiopulmonary resuscitation (6). Secondary hyperfibrinolysis further complicates the coagulation status of patients who have survived CA. This is evidenced by the elevated coagulation variables, fibrinolytic variables, and disseminated intravascular coagulation (DIC) scores observed in all patients with out-of-hospital CA who had measurements of activator XI-antithrombin complex, activator IX-antithrombin complex, and thrombin-antithrombin (TAT) complex, which serve as markers of coagulation activation. Additionally, increased coagulation variables, fibrinolytic variables, and DIC scores were associated with favorable neurologic outcomes. However, elevated DIC scores and mortality were also observed. An increase can be observed in correlation (11–14). It is unfortunate that thrombolytic drugs are associated with an improved prognosis. However, human trials have not found thrombolytic benefit (15–17).

In critical care, adjunctive circulatory support devices such as IABP, ECMO, CRRT and others often require the use of intravenous anticoagulants. However, upon discontinuing these devices and stopping intravenous anticoagulants, critical care physicians frequently face the question of whether to transition to oral anticoagulants. This decision is complicated by the risks associated with bleeding, particularly life-threatening hemorrhage and intracranial bleeding, which can significantly impact patient outcomes. As a result, most patients are not prescribed oral anticoagulants post-cardiac arrest unless they have pre-existing risk factors or a history of thrombotic events, such as pulmonary embolism, peripheral arterial thrombosis, or atrial fibrillation.

A meta-analysis evaluated the effect of additional intravenous anticoagulation or thrombolytic therapy in patients who had experienced cardiac arrest without ST-segment elevation on the electrocardiogram and had not undergone percutaneous coronary intervention (18). The analysis included two randomized controlled trials and one observational study. The findings indicated that intravenous anticoagulation or thrombolytic therapy was associated with an increased risk of bleeding without improving the time to return to spontaneous circulation or in-hospital mortality.

Similarly, a single-center observational study of 1,054 patients found that 295 (28%) had been on antiplatelet therapy prior to cardiac arrest, while 147 (14%) had been on anticoagulants. In the adjusted model, antiplatelet use was associated with lower disease severity scores and a higher likelihood of survival to hospital discharge post-cardiac arrest. In contrast, anticoagulant use did not correlate with disease severity, survival to discharge, or favorable outcomes (19). The anticoagulants studied included agents such as Alteplase, Argatroban, Bivalirudin, Apixaban, Dabigatran, Rivaroxaban, Dalteparin, Enoxaparin, Fondaparinux, Unfractionated Heparin, and Warfarin. Our data indicated a reduction in all-cause mortality from 28 days to one year among all patient subgroups who had ever used oral anticoagulants. However, due to inherent data limitations, we were unable to assess whether oral anticoagulant use provided added benefits in conditions associated with elevated thrombotic risk, such as acute myocardial infarction, ischemic stroke, and peripheral arterial thrombosis, or if it led to increased bleeding events. Additionally, because patients did not consistently adhere to long-term oral anticoagulant therapy and data on potential switches between different oral anticoagulants or overlap in use were unavailable, we could not compare the benefit-risk profiles of newer oral anticoagulants vs. warfarin within this cohort. This also precluded us from evaluating outcomes related to organ dysfunction, particularly the severity of coma. The MIMIC-IV database lacks granular temporal data (e.g., precise timestamps for cardiac arrest events) and does not systematically record in-hospital adjustments to anticoagulant therapy [e.g., discontinuation of oral anticoagulants (OAs) or transitions to parenteral agents like heparin]. This limitation hampers our capacity to isolate the independent impact of pre-arrest OA use from post-arrest management strategies, particularly for short-acting agents like direct-acting OAs. Our cohort was restricted to adult ICU patients with non-traumatic, non-surgical cardiac arrest—the target population for this study. While this selection strengthened internal validity by reducing confounding heterogeneity, it inherently limited the generalizability of our findings to other critical care contexts (e.g., out-of-hospital cardiac arrest, pediatric populations). Exclusions of short-stay ICU patients (<24 h), traumatic/post-operative cases, and individuals <18 years further narrowed the scope, prioritizing data quality over broad applicability. Although propensity score matching achieved standardized mean differences (SMDs) < 0.1 across all covariates, the observational nature of our study inherently limits causal inference. Unmeasured confounders—such as pre-arrest neurological status, socioeconomic factors, or variability in resuscitation protocols—may influence outcomes. Additionally, while PSM improved balance, it cannot fully account for dynamic clinical factors (e.g., in-hospital complications) that evolve post-arrest.

In summary, our findings suggest that oral anticoagulants may reduce survival rates to hospital discharge following cardiac arrest. Future studies should focus on characterizing clot burden in patients with cardiac arrest and identifying targets for early intervention to mitigate the disease progression following such events.

## Conclusion

In conclusion, our study suggests that oral anticoagulants may attenuate survival to hospital discharge after cardiac arrest.

## Data Availability

The original contributions presented in the study are included in the article/Supplementary Material, further inquiries can be directed to the corresponding author.
