# Supplementary material for: Pre–arrest oral anticoagulants’ impact on cardiac arrest mortality: MIMIC–IV cohort retrospect
Source: Front Cardiovasc Med. 2025 Nov 3;12:1599318. doi: 10.3389/fcvm.2025.1599318 (PMC12620496; doi:10.3389/fcvm.2025.1599318)
Supplement: Supplementary file 1 [file Table1.docx]

**Supplementary table S1.**Evaluation of predictive performance for the three models

| Event | Model I | | Model II | | Model III | |
| --- | --- | --- | --- | --- | --- | --- |
|  | AIC | BIC | AIC | BIC | AIC | BIC |
| 28 all-cause mortality | 8586.401 | 8591.492 | 7267.558 | 7406.527 | 5552.003 | 5853.281 |
| 90 all-cause mortality | 9713.507 | 9718.598 | 8249.106 | 8388.075 | 6428.942 | 6730.22 |
| 180 all-cause mortality | 9942.875 | 9947.965 | 8441.318 | 8580.287 | 6585.47 | 6886.748 |
| 365 all-cause mortality | 10286.28 | 10291.37 | 8766.718 | 8905.687 | 6873.109 | 7174.387 |

AIC, Akaike Information Criterion; BIC, Bayesian Information Criterion.
